# Cavitary Consolidation in Pulmonary Infarct Secondary to Embolism: A Rare Presentation

**DOI:** 10.7759/cureus.63644

**Published:** 2024-07-02

**Authors:** Mithun Nilgiri K., Rishi G Orakkan, Shailesh B Meshram

**Affiliations:** 1 Department of Respiratory Medicine, Dr. D. Y. Patil Medical College, Hospital and Research Centre, Dr. D. Y. Patil Vidyapeeth (Deemed to be University), Pune, IND

**Keywords:** deep vein thrombosis, pulmonary angiography, consolidation, rare presentation, anticoagulation, pulmonary embolism, pulmonary infarct, atypical presentation, radiographic features, cavity

## Abstract

Obstruction of the pulmonary artery or one of its branches, often due to thrombi from the deep veins of the lower extremities can result in a life-threatening condition known as pulmonary embolism. Pulmonary infarction, an unusual complication of pulmonary embolism occurs when the blood supply to lung tissue is obstructed, leading to tissue necrosis.

An 80-year-old man presented with a cough, breathlessness, and generalized weakness. He was vitally stable with no oxygen requirement, which could have suggested an infective etiology like pneumonia or tuberculosis. However, the presence of calf tenderness prompted us to perform a venous Doppler ultrasonography, which revealed deep venous thrombosis. This, combined with right atrial and ventricular dilation and moderate pulmonary artery hypertension observed on transthoracic echocardiography (2D ECHO), led us to recommend a CT pulmonary angiography. The angiography revealed an uncommon presentation of pulmonary embolism with multiple pulmonary infarcts.

Here, we chronicle an unusual case of pulmonary infarction secondary to pulmonary embolism, which presented radiologically as consolidation with an aseptic cavity, a rare and atypical triple occurrence.

## Introduction

A potentially fatal disorder like pulmonary embolism is brought on by a blood clot that travels to block one or more pulmonary arteries. These clots typically originate from the deep veins of the lower limbs. From an asymptomatic condition to severe cardiovascular and respiratory failure, this blockage impedes the blood flow to lung tissue, resulting in a wide spectrum of clinical symptoms. Pulmonary infarction, in which the affected lung tissue experiences necrosis as a result of lack of blood supply, is a serious but rather infrequent consequence of pulmonary embolism [1].

Diagnoses of pulmonary infarction can be difficult because of the wide range of clinical presentations that can occur and the frequent overlap with other respiratory illnesses like pneumonia, and tuberculosis. Pleuritic pain, hemoptysis, fever, and cough are symptoms that patients may present with, leading to an initial misdiagnosis.

The diagnosis might be made even more difficult by the fact that radiographic features such as peripheral consolidations, ground-glass opacities, and even cavitary lesions can resemble those of other illnesses like lung abscesses or tuberculosis [2].

Though infrequent, pulmonary infarction is a dangerous consequence that can worsen the clinical course of pulmonary embolism. The prognosis of patients with pulmonary infarction can be improved, and additional complications can be avoided with prompt identification and appropriate treatment.

We offer this case report in this context to emphasize how crucial it is to identify pulmonary infarction secondary to embolism in cases presenting with cavities and consolidation. In patients with unusual respiratory symptoms and a history suggestive of thromboembolic events, healthcare professionals must maintain a high degree of suspicion for pulmonary infarction.

## Case presentation

An 80-year-old male presented with generalized weakness, breathlessness, and cough of two weeks' duration, along with bilateral lower limb swelling for one month. He had no significant past medical history or exposure to illnesses like tuberculosis.

Physical examination showed pallor, bilateral pitting pedal edema, and bilateral inguinal hernia. On auscultation, crepitations were heard in the bilateral infra-axillary areas. He had a normal sensorium (Glasgow Coma Scale [GCS] score 15/15) and was vitally stable, with a pulse rate of 80 beats per minute, a respiratory rate of 20 breaths per minute, blood pressure of 110/80 mmHg, and peripheral oxygen saturation of 98% without supplemental oxygen.

**Figure 1:**
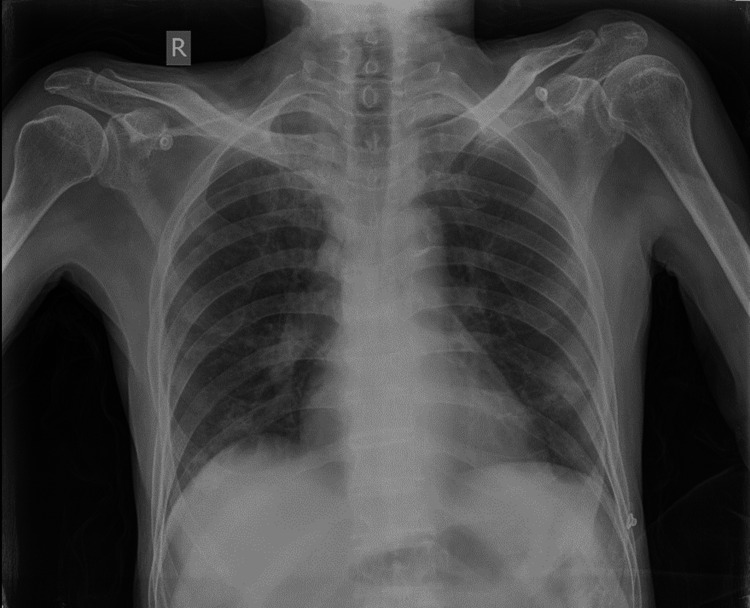
Chest radiograph showing no obvious abnormality.

The hemogram showed no significant derangements. His Well's score (for pulmonary embolism) was calculated to be 3.0, indicative of moderate risk for pulmonary embolism. D-dimer was found elevated to 1,722 ng/mL. A chest X-ray showed no obvious pleuroparenchymal abnormalities (Figure 1). Lower limb venous Doppler ultrasonography revealed thrombosis of the right popliteal vein, left common femoral vein, and left superficial femoral vein, and partial thrombosis of the left popliteal vein. The ECG revealed sinus rhythm with T inversion in the precordial leads (V2, V3, and V4). Transthoracic echocardiography (2D ECHO) showed an ejection fraction of 60%, moderate pulmonary artery hypertension, and mildly dilated right atrium and ventricle. Sputum Gram staining, Ziehl-Neelsen staining, and cultures yielded no positive results. Ultrasonography of the abdomen and pelvis indicated the presence of bilateral inguinal hernia.

**Figure 2:**
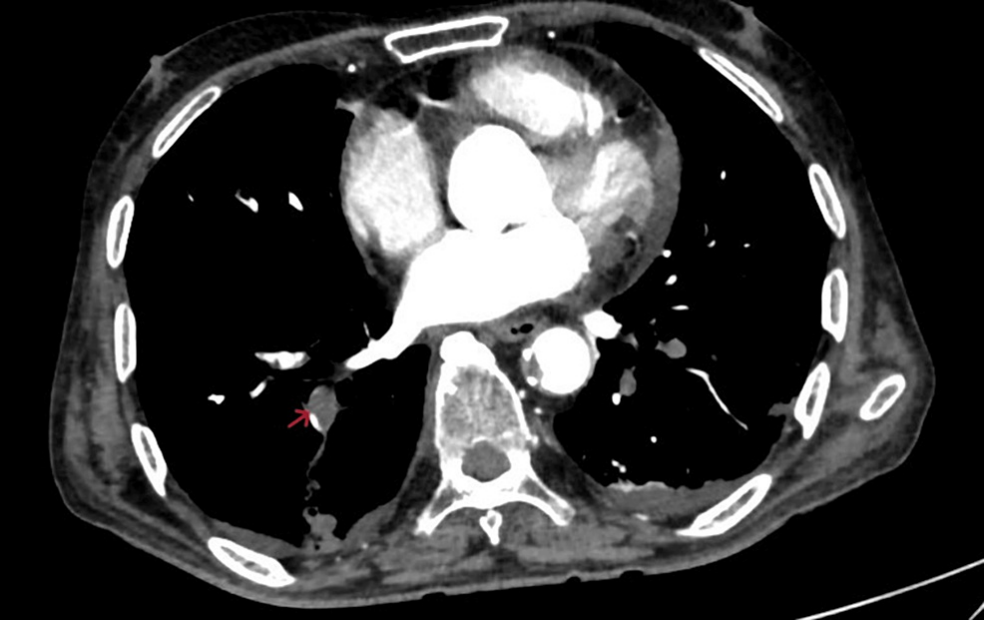
Axial CTPA showing a hypodense filling defect (red arrow) in the lower segmental branch of the right pulmonary artery. CTPA, computed tomography pulmonary angiography

**Figure 3:**
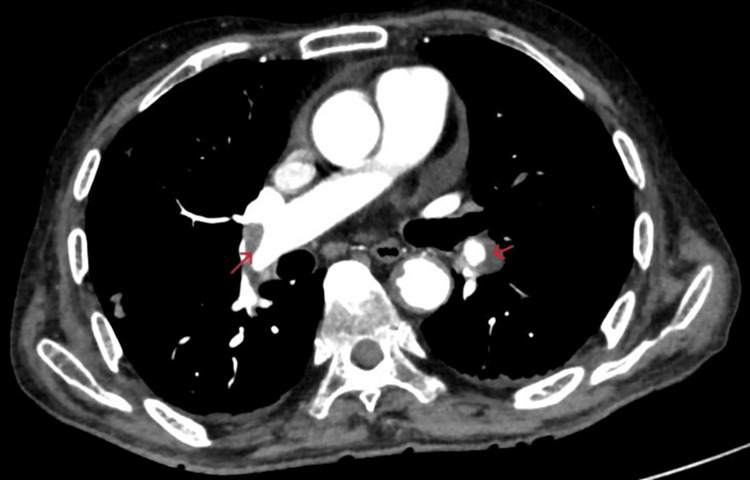
Axial CTPA showing hypodense filling defects (red arrow) in the right interlobar pulmonary artery and segmental branches of the left pulmonary artery. CTPA, computed tomography pulmonary angiography

Computed tomography pulmonary angiography (CTPA) was done, which showed a central filling defect noted along the anterior wall of the distal right interlobar artery extending into the right middle lobe segmental arteries, right superior segmental artery, and also in the inferior lobar artery in distal left main pulmonary artery extending into apicoposterior and anterior segmental artery and left interlobar artery (Figures 2-3).

**Figure 4:**
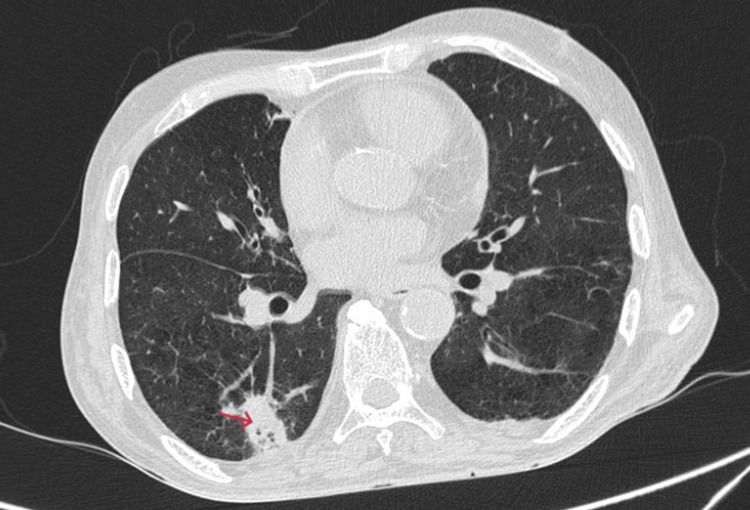
Axial CT lung window showing pulmonary infarct in the right lower lobe. CT, computed tomography

**Figure 5:**
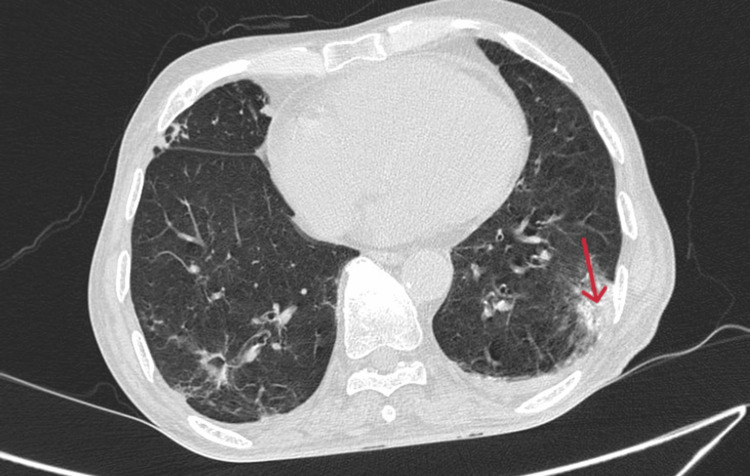
Axial CT lung window showing pulmonary infarct in the left lower lobe. CT, computed tomography

**Figure 6:**
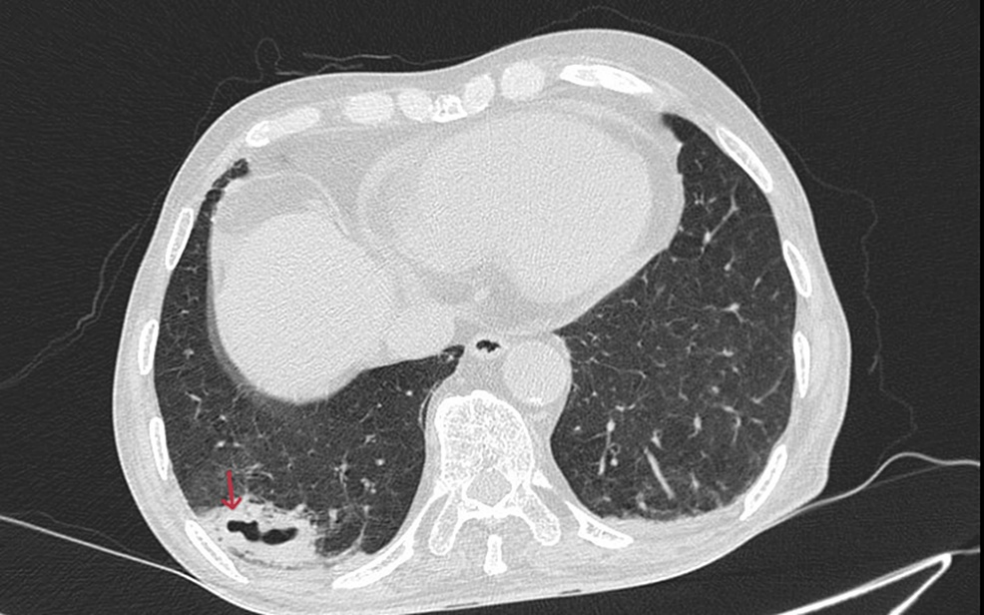
Axial CT lung window showing consolidation with cavitary lesions in the lower lobe of the right lung (red arrow). CT, computed tomography

Multiple ill-defined areas of peripheral consolidations with dense adjacent fibrotic infiltrates and ground-glass opacities were observed in the superior segment of the right lower lobe, the posterior and lateral basal segments of the right lower lobe, and the superior, anteromedial basal, and lateral segments of the left lower lobe. The features were suggestive of multiple bilateral pulmonary infarcts (Figures 4-5). Few of these consolidations in the posterior and lateral basal segments of the right lower lobe show cavitary breakdown (Figure 6).

The patient was initially treated symptomatically with antibiotics and nebulization with bronchodilators. Once diagnosed with pulmonary embolism and deep-vein thrombosis, prompt anticoagulation with injection of Enoxaparin sodium 60 mg (0.6 mL) subcutaneously twice daily was started. The patient was symptomatically better after initiation of administration of anticoagulants and thus discharged on oral anticoagulants (Rivaroxaban 15 mg twice daily).

## Discussion

Pulmonary embolism is a potentially fatal cardiovascular emergency, but its presentation can be highly variable. The occurrence of pulmonary infarction as a complication of pulmonary embolism is well-documented, though it remains relatively rare. Even more unusual is the presentation of pulmonary infarction with cavitation and consolidation, which can closely mimic other conditions such as pneumonia, tuberculosis, lung abscess, or even malignancies. Our case report illustrates this diagnostic challenge and the clinical significance of recognizing such atypical presentations [3].

This case involves an elderly man with extensive deep-vein thrombosis and multiple bilateral pulmonary infarcts concurrently presenting with cavitary consolidation. Therefore, in the differential diagnosis of any cavitating lung lesion, aseptic cavitation of a sterile pulmonary infarct should also be carefully evaluated [3].

Cavitation in the setting of pulmonary infarction occurs when necrotic lung tissue undergoes secondary infection or liquefaction, leading to the formation of cavities [4]. This process can be misleading, as it often resembles infectious processes in imaging studies.

Despite the initial presentation of cough, breathlessness, and generalized weakness, further investigations, including elevated D-dimer levels and lower limb venous Doppler ultrasonography findings of deep-vein thrombosis, along with transthoracic echocardiography (2D ECHO) showing a dilated right atrium and ventricle, raised suspicion of pulmonary embolism. Chest X-ray did not reveal any significant abnormalities. In our case, CTPA played a pivotal role in confirming our diagnosis of pulmonary embolism by revealing central and segmental filling defects in the pulmonary arteries, along with multiple areas of infarction.

The imaging findings of peripheral consolidations, fibrotic infiltrates, and ground-glass opacities were peculiar here. These features are indicative of an advanced pulmonary infarction with secondary changes, which, though uncommon, were clinically significant. The presence of cavities further complicated the clinical picture, as it necessitates differentiation from other potential causes such as bacterial or fungal infections, which can also present with similar radiologic findings [5], which we ultimately ruled out by various sputum-staining studies, sputum Cartridge-Based Nucleic Acid Amplification Test (CBNAAT), and sputum cultures.

Management of such complex cases requires a multidisciplinary approach. Anticoagulation remains the cornerstone of therapy for pulmonary embolism to prevent further thromboembolic events [6]. In cases of cavitary pulmonary infarction, additional considerations include the potential need for antibiotics if a secondary infection is suspected. They also require close monitoring for complications such as hemoptysis or pleural effusion. The role of imaging, particularly CT pulmonary angiography is crucial in the initial diagnosis [7].

This case underscores the importance of considering pulmonary infarction with cavitation and consolidation in the differential diagnosis of patients presenting with atypical respiratory symptoms and signs suggestive of peripheral thromboembolic disease. Early and accurate diagnosis is critical for appropriate management and significantly enhances patient outcomes. Clinicians should maintain a high index of suspicion for such rare presentations and utilize advanced imaging modalities to reach an accurate diagnosis. Further studies are necessary to enhance our understanding of the pathophysiology, optimize management strategies, and improve the prognosis of patients with such uncommon presentations of pulmonary embolism.

## Conclusions

A picture of cavitary consolidation might have led to a misdiagnosis of infectious etiology if a non-contrast CT had been performed on our patient. A detailed clinical examination led us to elicit calf tenderness in our patient, encouraging us to investigate deep-vein thrombosis and possible cardiac causes of breathlessness and further suggest a CT pulmonary angiography, finally leading us to an accurate diagnosis.

This case underscores the importance of considering pulmonary embolism and pulmonary infarction in differential diagnoses, even in the presence of misleading findings like cavitary consolidation. Early detection and appropriate management are essential for achieving the right diagnosis and improving patient outcomes. We highlight one such atypical presentation of cavitary consolidation in a case of pulmonary embolism with infarct.
